# Effect of violet LED light on in-office bleaching protocols: a randomized controlled clinical trial

**DOI:** 10.1590/1678-7757-2019-0720

**Published:** 2020-05-18

**Authors:** Matheus KURY, Erica Eiko WADA, Daylana Pacheco da SILVA, Cínthia Pereira Machado TABCHOURY, Marcelo GIANNINI, Vanessa CAVALLI

**Affiliations:** 1 Universidade Estadual de Campinas Faculdade de Odontologia de Piracicaba Departamento de Odontologia Restauradora PiracicabaSP Brasil Universidade Estadual de Campinas , Faculdade de Odontologia de Piracicaba , Departamento de Odontologia Restauradora , Piracicaba , SP , Brasil .; 2 Universidade Estadual de Campinas Faculdade de Odontologia de Piracicaba Departamento de Ciências Fisiológicas PiracicabaSP Brasil Universidade Estadual de Campinas , Faculdade de Odontologia de Piracicaba , Departamento de Ciências Fisiológicas , Piracicaba , SP , Brasil .

**Keywords:** Tooth Bleaching, Hydrogen Peroxide, Carbamide Peroxide, Light, Dentin Sensitivity

## Abstract

**Objective:**

This study evaluated the clinical effect of violet LED light on in-office bleaching used alone or combined with 37% carbamide peroxide (CP) or 35% hydrogen peroxide (HP).

**Methodology:**

A total of 100 patients were divided into five groups (n=20): LED, LED/CP, CP, LED/HP and HP. Colorimetric evaluation was performed using a spectrophotometer (ΔE, ΔL, Δa, Δb) and a visual shade guide (ΔSGU). Calcium (Ca)/phosphorous (P) ratio was quantified in the enamel microbiopsies. Measurements were performed at baseline (T _0_ ), after bleaching (T _B_ ) and in the 14-day follow-up (T _14_ ). At each bleaching session, a visual scale determined the absolute risk (AR) and intensity of tooth sensitivity (TS). Data were evaluated by one-way (ΔE, Δa, ΔL, Δb), two-way repeated measures ANOVA (Ca/P ratio), and Tukey post-hoc tests. ΔSGU and TS were evaluated by Kruskal-Wallis and Mann-Whitney, and AR by Chi-Squared tests (a=5%).

**Results:**

LED produced the lowest ΔE (p<0.05), but LED/HP promoted greater ΔE, ΔSGU and Δb (T _14_ ) than HP (p<0.05). No differences were observed in ΔE and ΔSGU for LED/CP and HP groups (p>0.05). ΔL and Δa were not influenced by LED activation. After bleaching, LED/CP exhibited greater Δb than CP (p>0.05), but no differences were found between these groups at T _14_ (p>0.05). LED treatment promoted the lowest risk of TS (16%), while HP promoted the highest (94.4%) (p<0.05). No statistical differences of risk of TS were found for CP (44%), LED/CP (61%) and LED/HP (88%) groups (p>0.05). No differences were found in enamel Ca/P ratio among treatments, regardless of evaluation times.

**Conclusions:**

Violet LED alone produced the lowest bleaching effect, but enhanced HP bleaching results. Patients treated with LED/CP reached the same efficacy of HP, with reduced risk and intensity of tooth sensitivity and none of the bleaching protocols adversely affected enamel mineral content.

## Introduction

Tooth bleaching is a common procedure in the office routine as aesthetic appeal is a permanent trend among patients. ^1^ Even though in-office bleaching gels are applied on tooth surface in a shorter period of time in comparison to those of at-home therapy, ^2^ a number of studies have attested the efficacy of in-office bleaching over the past decades. ^3^ ^,^ ^4^ Nevertheless, light activation of bleaching is a topic under constant discussion since a previous systematic review reported that light does not affect color change for high-concentrated hydrogen peroxide (HP) bleaching, but inconclusive results have been obtained for lower HP concentrations. ^5^ Conversely, Maran, et al. ^6^ (2017) stated that light did not enhance the efficacy of in-office bleaching regardless of the concentration of HP.

The impact of light activation on the adverse effects caused by tooth bleaching has also been investigated. ^5^ ^,^ ^7^ Light activation does not increase tooth sensitivity (TS) when high-concentrated hydrogen peroxide gel is used, and the concentration of peroxide itself does not seem to affect the prevalence and intensity of TS. ^6^ Nevertheless, a recent randomized clinical trial introduced 37% carbamide peroxide (CP) without light as a feasible alternative to reduce TS promoted by bleaching. ^8^ Furthermore, light activation does not modify the enamel morphology ^9^ nor exacerbate *in vitro* mineral changes, ^10^ it does not decrease calcium (Ca) and phosphorous (P) content nor increases surface roughness. ^11^ ^,^ ^12^ Even though the concentrations of Ca and P are clinically maintained after at-home and in-office bleaching, ^13^ there is no *in vivo* data on the effect of light-assisted in-office bleaching on the enamel mineral content.

In this context, a novel generation of violet LED light (LED) for in-office bleaching ^14^ has raised concerns on possible side effects due to the use of light as a bleaching protocol, ^15^ ^-^ ^17^ considering the lack of evidence supporting both its efficacy and safety. According to the manufacturer instructions, violet LED light should be used without bleaching gels in patients reporting moderate to intense TS, and LED could also be used with high-concentrated HP or CP in patients with absent or low TS. ^18^ Violet LED operates under an approximate 405 nm wavelength, ^14^ and it is speculated that its radiation presents the same absorbance peak of pigments on the enamel surface, causing a photolytic effect. ^15^ Recent *in vitro* studies demonstrated that a peroxide-free protocol with violet LED promoted color and whiteness changes in stained teeth. ^9^ ^,^ ^19^ Thus, it is expected that LED could prevent damages on enamel surface and the absence of HP and the diffusion of its by-products into the pulp chamber may reduce TS. On the other hand, the mechanism of action of violet LED combined with bleaching gels could be explained by the increase in gels temperature and, consequently, the increase in HP decomposition into free radicals, ^2^ thereby increasing bleaching efficacy.

Thus, this study aimed to assess efficacy (color change) of violet LED light in-office bleaching combined or not with high-concentrated peroxide gels (35% HP or 37% CP). Moreover, the effect of LED on tooth sensitivity and enamel mineral content were evaluated. The tested null hypotheses were that violet LED would 1) not promote the same color change as peroxide gels, 2) not enhance the efficacy of the bleaching gels, 3) not increase the risk and intensity of TS and 4) not cause changes to enamel mineral content when combined or not with CP or HP.

## Methodology

### Ethical approval and protocol’s registration

This clinical trial was approved by the local Research Ethics Committee (registration number: 2,294,061). The research was registered in the National Clinical Trials Registry (REBEC – RBR-5t6bd9).

### Trial design

This was a parallel, randomized, controlled and blind clinical trial, which followed the CONSORT guidelines. Patients were not aware of which treatment group they belonged to. A research member was responsible for randomizing the patients within the bleaching groups to ensure the allocation concealment mechanism. Although the clinical operator was informed of which group each participant was allocated to, the colorimetric analysis operator was blinded to the procedures.

### Recruitment and eligibility criteria

All patients included in this study signed an informed consent form, which is in accordance with the Declaration of Helsinki, before the first bleaching session. The volunteers were aged over 18 and under 60 years old, presenting no carious lesions and healthy gingival conditions. The eligibility requirements also included patients that had not undergone tooth bleaching over the last three years, and whose upper right canine minimum shade was A2 or darker.

The volunteers were excluded if they had one of the following conditions: enamel cracks, dentin hypersensitivity, extensive restorations, endodontically treated teeth and edentulous space between maxillary and mandibular premolars. Also, patients that would not be able to attend all the bleaching and follow-up appointments were excluded.

### Sample size calculation

Color change was the primary outcome of this study. A previous research ^20^ showed that a protocol with 35% HP agent without light activation resulted in 8.3±3.5 bleaching effect (ΔE). Based on that study, a 5% significance level and 80% power were used to calculate the minimum number of patients to detect differences between groups. According to the estimation (BioStat, AnalystSoft, Walnut, CA, USA), 16 patients per group would be required, but 20 patients per group were included, so that any possible volunteer dropouts would not affect the result.

### Randomization, allocation and blinding

Randomization was performed by a research member, who was not part of the bleaching and evaluation procedures. This person assigned a code to each participant. Each code was written in an opaque and sealed sheet, and sheets were distributed randomly among the five intervention groups. The result of this randomization was only revealed to the operator at the beginning of the first bleaching appointment. The participants were blinded to the procedure in terms of agent type (HP or CP) as they did not know which bleaching agent was applied on the tooth surface. Although volunteers treated only with LED irradiation could have noticed that no bleaching agent was applied, they were not informed about how their group differed from the others. The operator was aware of volunteers’ bleaching interventions since gels are visually different. The bleaching gel was not seen by the patients since an assistant supported the blind-procedure. The colorimetric rater was blinded to all procedures.

### Study intervention

**Figure 1:**
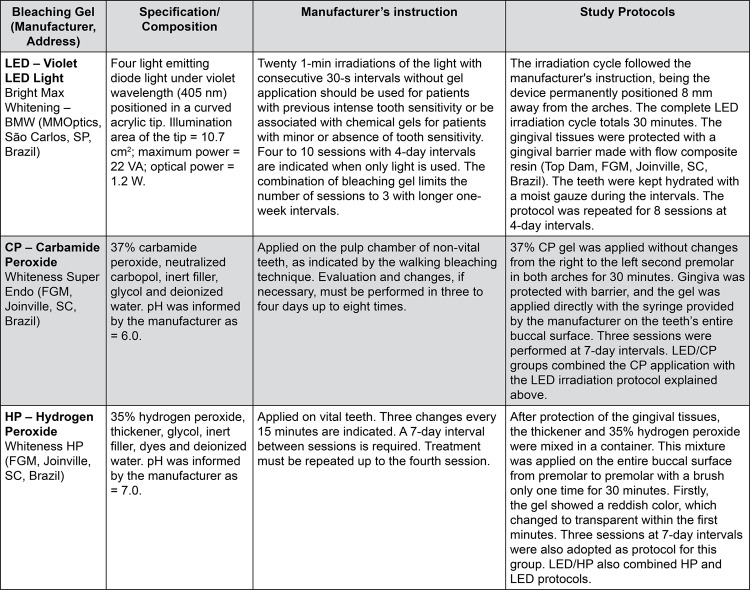
Bleaching gels, composition, light source, manufacturer’s instructions, and study protocols

Five different in-office bleaching protocols were defined as the interventions of this study. The groups were established according to each bleaching gel and light activation: (1) LED, (2) LED/CP, (3) CP, (4) LED/HP and (5) HP. The materials, light source and bleaching protocols are detailed in Figure 1 .

### Colorimetric evaluation

An objective evaluation of color change was conducted with contact-type intraoral spectrophotometer Easy Shade (Vita Zahnfabrik, Bad Säckingen, Germany). An impression with dense silicon (Zhermak, Kouigo, Italy) was preliminary obtained from volunteers’ upper right arch, and a hole with the same dimension of the spectrophotometer tip was created in the upper right canine. ^21^ Thus, the region of color analysis was standardized for all evaluation times. Dental prophylaxis was performed before the baseline color measurement and the patients were requested not to consume dark beverages and food. Before treatment (T _0_ ), the rater recorded the values of CIE L*a*b* coordinates, and this procedure was repeated after the last bleaching session (T _B_ ) and 14 days after the end of the intervention (T _14_ ). While L* represents luminosity, a* and b* indicate the measurement of the red*green and yellow*blue axes, respectively. ΔL, Δa and Δb were calculated at both time intervals: 1 (T _B_ -T _0_ ) and 2 (T _14_ -T _0_ ). Subsequently, the Δ values were individually applied in the formula ΔE= [(ΔL*) ^2^ +(Δa*) ^2^ +(Δb*) ^2^ ] ^1/2^ to obtain ΔE1 and ΔE2 for each specimen.

Additionally, a subjective color evaluation (ΔSGU) ^20^ was carried out by the same blind rater at the same evaluation times as the objective assessment, using a visual shade guide unit (Vita Zahnfabrik, Bad Säckingen, Germany). The guide tabs were sorted by value from highest (1) to lowest (16), as follows: B1, A1, B2, D2, A2, C1, C2, D4, A3, D3, B3, A3.5, B4, C3, A4 and C4. The color registered was subtracted from the initial shades, to calculate ΔSGU at T _B_ and T _14_ . The rater was calibrated to measure color at the middle third of the upper right canine.

### TS analysis

A visual scale was handed out to the volunteers at the end of each bleaching session. The volunteers were asked to spontaneously indicate the intensity of TS in each session using this scale ranging from 0 to 10, in which 0 was equal to no sensitivity and 10 to the most intense discomfort experienced by the volunteer. ^22^ The operator also asked the patients to record TS intensity between sessions. The patients were requested not to use dentifrices for reducing tooth sensitivity. The volunteers, who reported absence of TS during the periods of evaluations, indicated it as 0. When the patients reported it as at least level 1, they were considered to be sensitive to the intervention. Thus, the absolute TS risk for the bleaching protocols was measured.

### Enamel mineral content evaluation

An enamel microbiopsy was carried out to quantify calcium to phosphorus ratio of tooth submitted to interventions. In this study, the protocol published by Amaral, et al. ^13^ (2012) was adapted for use in the first upper premolar at T _0_ , T _B_ and T _14_ . The biopsy site was isolated by the operator with an adhesive tape (3M Oral Care, St. Louis, MN, United States) with a circular 1.6 mm perforation. Five μL of 1.6 M HCl in 70% glycerol (v/v) (Sigma-Aldrich, St. Louis, MO, United States) were applied in this region for 20 s with continuous gentle stirring. This solution was collected and dispensed in a test tube with 200 μL of ultra-purified water. Afterwards, 5 μL of 70% glycerol were applied on the same region for 10 s, and it was also transferred to the same tube.

The Arsenazo III and malachite green methods described by Vogel, et al. ^23^ (1983) were used to determine the Ca and P content in μg, respectively, in 25 μL of each sample. The absorbance was read in 96-well plates at 650 nm wavelength in a Multiskan Spectrum (Thermo Scientific, Waltham, MA, United States) microplate reader. The results were expressed as Ca/P ratio.

### Statistical analysis

The color change and mineral content data were submitted to exploratory analysis for normal distribution and homoscedasticity. The ΔL, Δa and Δb at both intervals met the parameters of normality, and ΔE was transformed into square root values after Levene’s test for equal variances. The Δ values were submitted to one-way ANOVA and Tukey’s Test. ΔSGU and intensity of the TS values were statistically analyzed using the Kruskal-Wallis and Mann-Whitney tests. The absolute TS risk was tested using non-parametric Pearson’s Chi Square test. The confidence interval for the absolute risk was estimated. The Ca/P ratio was tested using two-way repeated measures ANOVA (factors “ *treatment”* and “ *evaluation times* ”) and Tukey’s Test. The analyses were performed at 5% significance level, using SPSS Statistics Version 23 (IBM Corp, Armonk, NY, United States).

## Results

### Characteristics of the volunteers

**Figure 2:**
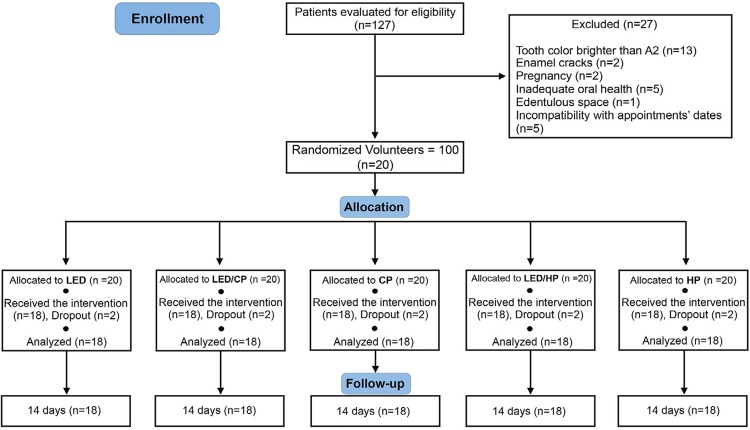
Flowchart of the RCT from the evaluation for eligibility of the volunteers to the

The recruitment occurred between November and December of 2017. Following the clinical examination for eligibility of 127 patients, 100 volunteers (n=20) were included in this study ( Figure 2 ). The characteristics and baseline values of the patients are described in Table 1 . The bleaching appointments were performed from January 15, 2018 to November 13, 2018. A dropout rate of 2 patients per group was detected. The reasons for dropout were incompatibility with the appointments dates or volunteers moving to distant cities. All patients (n=18) submitted to the intervention were evaluated for the primary outcome (color change) and for the secondary outcomes (TS and enamel mineral content).

**Table 1:** Baseline values for the CIEL*a*b* coordinates and main descriptive characteristics of the selected volunteers

		
Coordinate values (Mean, SD)	L*	81.84 (4.93)
	a*	5.96 (3.62)
	b*	27.13 (7.03)
Age in years (Mean, SD)		21.46 (4.14)
Gender (%)	Male	32.0%
	Female	68.0%
Ethnicity (%)	White	89.0%
	Black	5.5%
	Indigenous	2.2%
	Asian	3.3%

### Color change

Table 2 presents mean values and standard deviation of all parameters evaluated in each group. All protocols produced ΔE higher than 1.2. LED/HP treatment promoted the highest ΔE at both intervals (p<0.001) followed by HP and LED/CP, which exhibited no differences in ΔE (p>0.05). At both intervals, HP produced greater ΔE than CP (p<0.05). LED/CP showed higher ΔE than CP at ΔE1, but 14 days after bleaching (ΔE2), no differences were found. Patients treated only with LED showed the lowest ΔE at both intervals (p<0.05). LED group exhibited the lowest ΔSGU1 results (p<0.05), but 14 days after bleaching, no differences in ΔSGU2 were observed for LED and CP groups (p=0.97). LED/HP produced the highest ΔSGU at both intervals (p<0.05).

**Table 2:** Mean ΔE, ΔL, Δa and Δb1 (T B – T 0 ) and 2 (T 14 – T 0 ) values and standard deviations, and medians (minimum and maximum values) of ΔSGU 1 (T B – T 0 ) and ΔSGU 2 (T 14 – T 0 ), according to the treatments

Treatments	ΔE 1 (T _**B**_ – T _**0**_ )	ΔE 2 (T _**14**_ – T _**0**_ )
LED	3.4 (1.3) D	3.7 (1.4) D
LED/CP	7.8 (2.0) B	8.6 (2.1) BC
CP	5.7 (2.5) C	6.6 (3.0) C
LED/HP	12.9 (2.6) A	14.4 (2.2) A
HP	8.8 (3.0) B	10.0 (4.1) B
**Treatments**	**ΔSGU 1 (T**_**B**_ **– T**_**0**_ **)**	**ΔSGU 2 (T**_**14**_ **– T**_**0**_ **)**
LED	3.5 (0.3;4.0) C	2.5 (1.0;4.0) C
LED/CP	7.0 (6.0;8.8) B	6.0 (4.5;7.0) B
CP	5.5 (3.0;7.0) B	3.0 (3.0;6.0) BC
LED/HP	10.0 (7.0;10.0) A	9.0 (7.0;10.0) A
HP	7.0 (4.5;8.5) B	7.0 (4.5;7.8) B
**Treatments**	**ΔL 1 (T**_**B**_ **– T**_**0**_ **)**	**ΔL 2 (T**_**14**_ **– T**_**0**_ **)**
LED	-0.3 (2.5) C	-1.0 (1.7) D
LED/CP	2.9 (3.2) BC	2.6 (3.6) BC
CP	3.4 (2.8) B	4.5 (3.2) B
LED/HP	8.0 (3.4) A	7.7 (3.3) A
HP	5.0 (3.0) AB	5.6 (3.2) AB
**Treatments**	**Δa 1 (T**_**B**_ **– T**_**0**_ **)**	**Δa 2 (T**_**14**_ **– T**_**0**_ **)**
LED	0.0 (1.19) A	0.34 (0.7) A
LED/CP	-0.9 (1.8) AB	-1.0 (1.0) AB
CP	-1.6 (1.2) BC	-1.7 (1.6) BC
LED/HP	-2.0 (1.5) BC	-1.9 (1.3) BC
HP	-2.7 (1.0) C	-2.5 (1.6) C
**Treatments**	**Δb 1 (T**_**B**_ **– T**_**0**_ **)**	**Δb 2 (T**_**14**_ **– T**_**0**_ **)**
LED	0.0 (2.3) B	-1.1 (2.5) C
LED/CP	-6.0 (2.4) A	-6.3 (2.3) B
CP	-2.3 (2.1) B	-3.6 (2.5) B
LED/HP	-8.2 (4.2) A	-11.1 (5.1) A
HP	-6.2 (2.5) A	-7.2 (2.5) B

Uppercase letters compare bleaching protocols (treatments).

LED was the only group that did not increase teeth luminosity (ΔL). LED/HP and LED/CP did not increase ΔL and Δa of HP and CP, respectively (p>0.05). LED and CP (p=0.128) presented no differences in Δb1 but were significantly different after 14 days (Δb2; p=0.055). No differences in Δb1 were observed for LED/HP, HP and LED/CP treatments (p>0.05), but LED/HP showed the highest Δb2 (p<0.05) and LED presented the lowest Δb2 (p<0.05).

### TS

TS values are presented up to the third session because patients under LED treatment reported no TS after the third appointment. Regarding the absolute risk of TS ( Table 3 ), patients were considered sensitive even if they reported sensitivity at least in only one bleaching session. The Chi-square test revealed statistically significant differences between groups (p<0.001). Patients submitted to LED bleaching presented the lowest risk of TS (16%) and HP treatment promoted the highest risk of TS (94.4%) (p<0.05). No statistically significant differences were found for CP (44%), LED/CP (61%) and LED/HP (88%) groups (p>0.05), but the absolute number of patients with risk of TS treated with LED/HP (16) was higher than those treated with LED/CP (11) and twice the number of patients under CP treatment with risk of TS (8). The intensity of TS was evaluated at each session and intervals ( Table 4 ). At the 1 ^st^ session, patients submitted to LED and LED/CP treatments exhibited lower intensity of TS than those of LED/HP and HP groups (p<0.05). At the 2 ^nd^ session, no differences in intensity of TS were found among groups (p>0.05). During the 1 ^st^ interval and at the 3 ^rd^ session, LED/HP-treated patients reported more intensity of TS than those of HP group (p<0.05). During the 2 ^nd^ and 3 ^rd^ intervals, no differences were found for HP and CP and their respective LED light activated groups (p>0.05).

**Table 3:** Number of patients with and without tooth sensitivity (TS) and risk of TS of each bleaching protocol with corresponding 95% confidence interval

Treatments	Number of Patients with TS	Number of Patients without TS	Absolute Risk (95% Confidence Interval)
LED	3	15	0.16 (0.00-0.32)
LED/CP	11	7	0.61 (0.40-0.81)
CP	8	10	0.44 (0.23-0.65)
LED/HP	16	2	0.88 (0.74-1.04)
HP	17	1	0.94 (0.84-1.06)

Pearson’s Chi square test (p<0.001)

**Table 4:** Median values (minimum and maximum value) of tooth sensitivity (TS) intensity reported by the patients during the sessions and intervals

Treatments	1 ^**st**^ session	2 ^**nd**^ session	3 ^**rd**^ session
LED	0.0 (0.0;4.0) B	0.0 (0.0;4.0) A	0.0 (0.0;2.0) B
LED/CP	0.0 (0.0;3.0) B	0.0 (0.0;3.0) A	0.0 (0.0;7.0) AB
CP	0.0 (0.0;3.0) AB	0.0 (0.0;10.0) A	0.0 (0.0;4.0) B
LED/HP	2.0 (0.0;7.0) A	1.0 (0.0;9.0) A	2.0 (0.0;7.0) A
HP	2.5 (0.0;3.0) A	0.0 (0.0;7.0) A	0.0 (0.0;3.0) B
**Treatments**	**1** ^**st**^ **interval**	**2** ^**nd**^ **interval**	**3** ^**rd**^ **interval**
LED	0.0 (0.0;1.0) C	0.0 (0.0;3.0) C	0.0 (0.0;7.0) B
LED/CP	0.0 (0.0;5.0) BC	0.0 (0.0;6.0) B	0.0 (0.0;6.0) AB
CP	0.0 (0.0;5.0) C	0.0 (0.0;6.0) B	0.0 (0.0;3.0) B
LED/HP	5.0 (0.0;9.0) A	3.0 (1.0;7.0) A	3.0 (0.0;7.0) A
HP	0.5 (0.0;10.0) B	2.0 (0.0;10.0) AB	0.0 (0.0;10.0) AB

Medians followed by different letters differ statistically at 5% significance level, according to the Kruskal-Wallis and Mann-Whitney tests.

Uppercase letters compare TS according to treatments and to the sessions’ intervals. No TS was reported for the LED group after the third session.

### Enamel mineral content

Table 5 illustrates Ca/P ratio results according to treatments and evaluation times. The factor “treatment” presented statistical differences (p=0.009), but no difference was found for the factor “evaluation times” (p=0.654). The Ca/P ratio of CP was significantly higher than LED/CP and LED/HP at T _0_ , and no differences were detected among LED/HP, LED/CP, HP and LED (p>0.05). After bleaching (T _B_ ), CP and LED exhibited higher Ca/P ratio than LED/HP, and the higher Ca/P ratio of CP compared to LED/HP, was maintained even 14 days after bleaching (T _14_ ). The Ca/P ratio did not decrease (p>0.05) at each evaluation time, except for LED/CP that increased Ca/P concentration 14 days after bleaching (T _14_ ) compared to (T _B_ ) but remained with mineral content similar to T _0_ .

**Table 5:** Mean values and standard deviations of the Ca/P ratio according to treatments and evaluation times

Treatments	Ca/P (T _**0**_ )	Ca/P (T _**B**_ )	Ca/P (T _**14**_ )
LED	2.33 (0.88) ABa	1.98 (0.68) Aa	1.96 (0.80) ABa
LED/CP	1.76 (0.98) Bab	1.59 (1.12) ABa	2.30 (1.52) ABb
CP	2.65 (1.38) Aa	2.37 (1.59) Aa	2.49 (1.02) Aa
LED/HP	1.79 (0.94) Ba	1.40 (0.88) Ba	1.68 (1.07) Ba
HP	2.14 (0.71) ABa	1.85 (0.87) ABa	2.19 (1.13) ABa

Means followed by different letters differ statistically at 5% significance level, according to two-way repeated measures ANOVA and Tukey Test.

Uppercase letters compare treatments and lowercase letters compare evaluation times.

T _0_ (baseline), T _B_ (after bleaching) and T _14_ (14 days after bleaching).

## Discussion

The introduction of a new generation of violet LED for in-office bleaching and the suggestion of its association with peroxide gels raise concerns over this treatment safety and efficacy. Case reports, which applied violet LED without any chemical agent, showed color changes according to the visual shade guide after approximately 5 sessions. ^15^ ^,^ ^16^ In a study conducted *in vitro* , Gallinari, et al. ^19^ (2019) concluded that the use of violet LED light alone resulted in perceivable color change, but bleaching results were less effective than those promoted by HP-gels. Similarly, our study showed that LED protocol reached values above the clinically noticeable difference of 1.2 units for ΔE **,** ^24^ but LED alone promoted the lowest color change at both intervals among the groups. The individual analyses of the L*, a* and b* coordinates revealed that while luminosity (ΔL) was not affected by violet LED alone, the reduction of yellow appearance (Δb) at time point T _14_ played a role in color change for the LED protocol. This could be explained by the violet light wavelength, which is approximately 405 nm. The emission band of the violet LED is believed to correspond to the absorption peak of the stained particles, which leads them to decompose into shorter and uncolored molecules. ^14^ As pigments are reactive to light and violet light has lower capability of penetration through teeth, ^25^ we believe that the LED mechanism when applied alone is restricted to the enamel surface. Dental prophylaxis was performed at the beginning of each session to reduce extrinsic staining, and to not overestimate the color change produced by LED compared to the other groups. Moreover, the decision of interrupting the LED protocol after 8 sessions was based on the fact that no color change was detected after the 6 ^th^ appointment.

The subjective evaluation (ΔSGU) indicated similar color change between LED and CP 14 days after bleaching. Nevertheless, this was not observed for objective evaluation (ΔE) of CP, which exhibited higher ΔE than LED (p<0.05). Based on these results, the first null hypothesis that LED would not result in the same color change as gel protocols was rejected. Despite the fact that the rater was calibrated and the illumination conditions were standardized throughout the study, differences between ΔSGU and ΔE may have been caused by the relevant characteristics of each evaluation method. According to Joiner and Luo ^26^ (2017), visual shade guides have some drawbacks, such as inadequate range of shades and systematic inconsistencies between shade tabs. On the other hand, clinical studies verified great accuracy and repeatability of the spectrophotometer, e.g., VITA Easyshade. ^27^ ^-^ ^29^

At time point T _B,_ LED/CP promoted greater effectiveness than CP. This difference might be explained by a greater Δb promoted by LED/CP compared to CP at same interval. This could lead to the assumption that violet LED enhanced the rate of carbamide peroxide decomposition into free radicals, possibly caused by the heat, and the by-products were able to interact with the organic chromophores of dentin. ^30^ However, the Δb difference between these groups was no longer detected 14 days after bleaching. It is suggested that a residual effect may occur, since CP presents prolonged decomposition rate, ^31^ which could explain why the effectiveness of LED/CP and CP were similar at T _14._ However, similar color change was observed between the LED/CP and HP treatments with lower sensitivity levels for LED/CP. Considering this fact, the light activation of CP could be an alternative for patients with moderate-intense tooth sensitivity.

There is a lack of *in vitro* studies on the efficacy and safety of the application of high-concentrated carbamide peroxide. Peixoto, et al. ^8^ (2018) reported, in a randomized clinical trial, that color change resulted from bleaching with 37% CP was significantly lower than that with 35% HP, which corroborates our results. On the other hand, we showed that light activation overcame this limitation and it maintained the benefits for TS response. Although no randomized clinical trials tested the effects of light on 37% CP, several studies reported different findings regarding the efficacy of light activation for low-concentrated HP gels, whose hydrogen peroxide concentration is similar to that of 37% CP. ^2^ Thus, concentration may not be the only factor to affect bleaching efficacy. The long-term follow-up of color change outcomes for LED/CP will help to elucidate whether its efficacy is comparable to bleaching with high-concentrated HP over time.

The use of violet LED significantly enhanced the efficacy of HP even under the 14-day subjective and objective color evaluation, thus invalidating the second null hypothesis that LED would not enhance the efficacy of bleaching gels. The ability of LED/HP to decrease yellow appearance (Δb) was statistically superior to HP only at T _14_ (p<0.05), which suggests a residual effect of light-activated HP. Increase in gel temperature by this specific protocol, leading to extended formation of by-products, could have prolonged bleaching action of HP gel. A systematic review indicated that the bleaching efficacy of low and high-concentrated HP gels is not influenced by light activation. ^6^ Nevertheless, only few studies evaluated the use of light sources with a violet light wavelength component, and the activation time of LED, laser and halogen lamps differed remarkably among studies. For instance, while Kugel, et al. ^32^ (2009) irradiated 25% HP with a non-specified light source during the 60-minute gel application, Freitas, et al. ^33^ (2016) applied LED/laser for 3 minutes to activate 35% HP. Moreover, a recent network meta-analysis demonstrated that no light source presented superiority of color change outcomes. ^34^ Although we acknowledge the contribution of these systematic reviews for this field of study, the generation of violet LED should be considered as a new approach to in-office bleaching, and further studies on its efficacy should test its irradiation protocols, which, up until this moment, have only been established by the manufacturer. Just as for LED/CP, the follow-up of LED/HP will be helpful to determine its long-term efficacy.

The suggestion of new clinical protocols also considers tooth sensitivity levels, a meaningful safety parameter reported by patients. Several *in vivo* clinical trials have shown TS as a common side effect of tooth bleaching. ^6^ ^,^ ^21^ ^,^ ^35^ Even though our sample size was not calculated for the TS evaluation and these data should be evaluated cautiously, this secondary outcome showed that the absolute risk for LED/HP was lower than for HP, which ensures that light activation does not affect the chance of provoking TS in patients treated with HP. However, LED intensified the TS in these patients during the first week-interval and the last session, which would be a limitation of the LED/HP protocol. Also, the number of patients that reported TS for CP increased under LED activation (LED/CP>CP). Thus, the third null hypothesis was rejected, as LED affected patients’ TS. Nevertheless, it is noteworthy that LED/CP led to lower risk of TS than HP, with same color outcomes, which reassures that LED/CP might be an alternative for patients seeking for in-office treatments with reduced tooth sensitivity.

The 30-s intervals during the irradiation cycle of violet LED are performed as an attempt to not overheat the pulp and, consequently, not cause irreversible damage to the tissue. Although studies on the temperature rise of light-assisted bleaching are controversial in terms of which light source raises the temperature the most, ^36^ ^,^ ^37^ an *in vivo* research showed that a 60-s polywave LED unit irradiation for light-curing increased pulp temperature over 5.5 °C, ^38^ which could result in significant pulp necrosis. ^25^ Yet, no data were found to ensure whether the time interval indicated by the violet LED manufacturer is adequate or if a longer cooling phase should be adopted to protect pulp tissue even in the absence of bleaching gel. Further investigations should be performed on the safety of the protocols, such as pulp temperature and cell viability, and as the color change after HP application alone is considered extremely clinically perceptible, ^24^ this should be considered in the decision of using light activation of this gel.

Enamel microbiopsy is a technique used to clinically detect the concentration of ions such as fluoride, calcium and phosphorus on the surface, and it analyzes mineral changes after different challenges. ^13^ Even though the Ca/P ratio was different between some groups throughout the study, this ratio was maintained within each group between T _0_ and T _14_ . Therefore, the last null hypothesis was accepted. The inherent variation of the enamel mineral content ^13^ could explain the differences between patients at baseline. As LED did not alter the Ca/P ratio after the application of HP and CP, the irradiation of violet light does not seem to harm enamel structure or enhance the effect of the gel on the mineral content. An *in vitro* study found that 38% HP without light activation was able to change the conformation of the enamel prisms and interprismatic spaces. ^39^ Nonetheless, this pattern was not found for the same bleaching protocols tested *in situ* . Therefore, the presence of salivary pellicle may protect enamel surface against changes promoted by the bleaching gel application. Furthermore, the same authors stated that pH plays an important role in the enamel surface changes *in vitro.* ^39^ Contrariwise, Pinto, et al. ^12^ (2017) observed that a bleaching agent with acidic pH did not cause changes in the enamel mineral content. Therefore, the choice of the bleaching agent to be used in combination with violet LED should also consider the agent rheology and composition.

According to the results, violet LED light with or without the gels was efficient in terms of color change and did not influence enamel mineral content, but application of LED alone results in lower bleaching outcomes. The decision on which gel is more adequate will depend on the patient’s expectancy and initial color measurement, since LED/HP resulted in greater color change. Moreover, tooth sensitivity should be considered since, although LED adversely influenced TS of both bleaching gels separately, the LED/CP protocol reached the efficacy of HP group with lower TS absolute risk. The bleaching protocol should be performed after correct tooth discoloration diagnosis. Furthermore, patients must be aware that color change with LED alone will not reach similar efficacy to that of chemical gels, and the treatment regimen is longer.

## Conclusion

Violet LED light alone promoted a clinically perceptible color change, but it did not reach the same efficacy as the HP-treated groups. The association of LED with HP enhanced color change, and light activation of high-concentrated CP led to similar efficacy of HP, with lower tooth sensitivity. Violet LED activation of in-office bleaching protocols did not adversely affect mineral content on enamel surface.
